# Preliminary Studies on Graphene-Reinforced 3D Products Obtained by the One-Stage Sacrificial Template Method for Bone Reconstruction Applications

**DOI:** 10.3390/jfb12010013

**Published:** 2021-02-12

**Authors:** Aura-Cătălina Mocanu, Florin Miculescu, George E. Stan, Robert-Cătălin Ciocoiu, Mihai Cosmin Corobea, Marian Miculescu, Lucian Toma Ciocan

**Affiliations:** 1 Department of Metallic Materials Science, Physical Metallurgy, University Politehnica of Bucharest, 313 Splaiul Independentei, J Building, RO-060042 Bucharest, Romania; mcn_aura@hotmail.com (A.-C.M.); ciocoiurobert@gmail.com (R.-C.C.); m_miculescu@yahoo.com (M.M.); 2 National Institute of Materials Physics, 405A Atomistilor Street, RO-077125 Măgurele, Romania; george_stan@infim.ro; 3 Polymers Department, National Institute for Research & Development in Chemistry and Petrochemistry, 202 Splaiul Independentei, RO-060021 Bucharest, Romania; mcorobea@yahoo.com; 4 Prosthetics Technology and Dental Materials Department, “Carol Davila” University of Medicine and Pharmacy, 37 Dionisie Lupu Street, RO-020022 Bucharest, Romania; tciocan@yahoo.com

**Keywords:** marble, graphene, biogenic-calcium-phosphate, natural template, reinforced products, mechanical features

## Abstract

The bone remodeling field has shifted focus towards the delineation of products with two main critical attributes: internal architectures capable to promote fast cell colonization and good mechanical performance. In this paper, *Luffa*-fibers and graphene nanoplatelets were proposed as porogen template and mechanical reinforcing agent, respectively, in view of framing 3D products by a one-stage polymer-free process. The ceramic matrix was prepared through a reproducible technology, developed for the conversion of marble resources into calcium phosphates (CaP) powders. After the graphene incorporation (by mechanical and ultrasonication mixing) into the CaP matrix, and *Luffa*-fibers addition, the samples were evaluated in both as-admixed and thermally-treated form (compact/porous products) by complementary structural, morphological, and compositional techniques. The results confirmed the benefits of the two agents’ addition upon the compact products’ micro-porosity and the global mechanical features, inferred by compressive strength and elastic modulus determinations. For the porous products, overall optimal results were obtained at a graphene amount of <1 wt.%. Further, no influence of graphene on fibers’ ability to generate at high temperatures internal interconnected-channels-arrays was depicted. Moreover, its incorporation led to a general preservation of structural composition and stability for both the as-admixed and thermally-treated products. The developed CaP-reinforced structures sustain the premises for prospective non- and load-bearing biomedical applications.

## 1. Introduction

Dentistry and orthopedic surgery advancements outlined a new, challenging, and necessary era for bone loss reconstruction techniques involving novel biomaterials and products. Tissue engineering and the regenerative medicine field continuously focus on restoring both the form and function in the event of integrity discontinuities or tissue failure. For bone tissue, this strategy comes as a result of the bone’s limited capacity to repair itself (apart from small skeletal fractures). Therefore, extensive bone defects that are above the critical size and result from accidents, trauma impact, congenital malformations, surgical resections, or bone diseases require filling treatment techniques [1]. Furthermore, with the aging of the population and the increase in life expectancy, the number of patients with osteoporosis will naturally grow [1,2,3].

The bone regeneration itself is a complex, temporal, and spatial process, meant to render the bone in a form that is difficult to differentiate from the initial state [4]. Currently, the gold standard for bone reconstruction is autografting/autologous bone grafting (bone tissue harvesting from different body parts of the patient) [5]. Given the several downsides of these processes, including morbidity, supplementary surgery, and reduced bone graft quantities [1], these grafting solutions prove to be also incompatible with the large-area bone defects repair. As an alternative, bone synthetic grafts/scaffolds based on biocompatible materials are developed. [6,7,8,9,10].

Calcium phosphate (CaP)-based materials stirred up interest for almost three decades due to their biomimicry, biocompatibility, and osteoconductive properties [11,12]. They are now in high demand as reconstruction materials due to the recent technological advancements which allow for their industrial production [13,14]. Furthermore, the current trends promote the bio-functionalization of natural resources as an eco-friendly, cost-efficient, and sustainable alternative for their synthesis [15,16,17,18].

Nature offers a wide range of materials with great potential for this area or research, including sustainable resources of geographically widespread calcium carbonate. It covers more than 4% of the total surface of the Earth, being found both in the land and marine environments [19]. In this regard, marble and seashell resources can now be converted into biocompatible hydroxyapatite (HA, Ca_10_(PO_4_)_6_(OH)_2_) or biphasic HA/brushite (CaHPO_4_·2H_2_O) which can decompose at high temperatures into biphasic HA/β-tricalcium phosphate (β-TCP, Ca_3_(PO_4_)_2_) with a tunable ratio via an recently established, facile, sustainable, and reproducible synthesis route, as reported elsewhere [16,20]. Furthermore, the cost-efficiency issues are also curtailed by the abundance of source material resources, and consequently the technical aspects regarding the correction of large bone defects (with emphasis on the significant quantity of material necessary to accomplish such an objective) could be easily tackled [1,18,21].

It is stated that an ideal 3D structure destined for skeletal repair applications should mimic the biological, compositional, and mechanical properties of the host bone and also create the necessary niche for further functionalization [7,22]. Although micro-porosity is important for the bioresorbability of the material, and a high macro-porosity facilitates revascularization and growth of bone tissue inside the scaffold, the major disadvantage that occurs in the case of a too large pore volume is the reduction of mechanical strength. Therefore, an advantageous compromise between the porosity and mechanical response is necessary to ensure a suitable in vivo performance [23].

One way to address this aspect is to design scaffold architectures with large pores/channels by mixing ceramic materials with volatile materials, polymeric porogens [7], or natural fibers [23,24]. The compatible-use of *Luffa* fibers as porous template and its beneficial influence on the structure and phase composition of the ceramic matrix prior- and post-sintering, along with the influence of compressing force on the existence and importance of a secondary micro-porosity induced on internal channel surfaces, were recently reported and thoroughly discussed [8,25]. It was concluded that the use of vegetal fibers allows for the development of a vascular-like network through which the oxygen and nutrients transport—crucial for cell viability [26]—is warranted. In natural bone tissue, these functions are provided by a highly branched system of larger blood vessels, which is subdivided into small capillaries [7]. The obtained porous structures with interconnected channels are a result of fibers calcination, also known as the ‘sacrificial template/porogen method’, and the micro-porosity is a consequence of temperature and sintering duration [8,25].

On the other hand, lately, graphene-based materials gathered much attention in the biomedical realm (i.e., being included in implant coatings or used as a composite reinforcement phase), due to their capability to increase the mechanical resistance, elasticity, and flexibility and to generate antibacterial effects [3,27,28,29,30]. The possibility to incorporate such materials into a ceramic matrix emerged only a few years ago; several chemical synthesis routes were reported, but these can be time-consuming, expensive, and still insufficiently improved in terms of reproducibility [31,32]. Moreover, the associated biological response is dictated by the chemical, morphological, and structural properties of graphene. In this regard, graphene architectures with two micrometric dimensions and only one nanometric dimension could improve the biofunctionality as compared to the frequently investigated nano-sized graphene materials, since the cell viability is mainly size-dependent and better promoted by materials with lower overall surface areas [27,33].

The aim of this study is to develop compact and porous 3D products by the incorporation/admixing of graphene nanoplatelets and *Luffa* fibers into calcium phosphate matrices for future implementation in both non- and load-bearing bone regeneration applications. This way the chemical processing routes or the use of polymeric binder materials was avoided. The ceramic matrix synthesis will follow the aforementioned reproducible technology [16,20] and the porous configuration will be generated through the sacrificial template method [8,25]. Given the well-known inter-play between all mentioned aspects, several key-points related to the tailored material and 3D structures characteristics will be clarified in this work: (i) the optimum graphene nanoplatelets amount for an adequate morphological, compositional, and mechanical properties; (ii) the graphene distribution and incorporation level within the ceramic matrix; and (iii) the influence of high sintering temperature on the mixed natural templates and graphene materials for scaffolding processing. Therefore, our efforts will be directed towards the proper delineation of the graphene addition with respect to the required/adequate porosity and mechanical features for both compact and porous products in view of a favorable bone osseointegration.

## 2. Materials and Methods

### 2.1. Sample Preparation

The fabrication of the reinforced bioceramic-based 3D products starts with the CaP powder synthesis. This step is achieved based on the conversion of dolomitic marble through an already established, facile, and completely reproducible route, as previously reported [8,16,25]. Briefly, the process is constituted by the thermal decomposition of calcium carbonate into calcium oxide (CaO) at 1300 °C/6 h, and the further hydration for calcium hydroxide powder formation (Ca(OH)_2_), followed by chemical treatment with phosphoric acid (H_3_PO_4_, 85%, Sigma Aldrich, St. Louis, MO, USA) at stoichiometric ratios—200 mL distilled water, 10 g of Ca(OH)_2_, and 5.5 mL of H_3_PO_4_ (magnetic stirring for 2 h at 25 °C, acid addition rate of 1 mL/min). After the drying treatment (100 °C/2 h) in autoclave (SN30, Memmert GmbH + Co. KG, Schwabach, Germany), the synthesized powders were deposited in Petri dishes and sealed in a desiccator.

Graphene nanoplatelets (Gr), grade M (XG Sciences, Lansing, MI, USA) with an average thickness of approximately 6–8 nm and average particle diameter of 5 μm, were incorporated in the ceramic powder by mechanical mixing (Inversina-2L-manual, Bioengineering AG, Wald, Switzerland) for 30 min, followed by dispersion with an ultrasonication probe (SONICS Vibra Cell, Sonics & Materials, Inc., Newtown, CT, USA) for another 30 min. Amounts of Gr were incorporated into the samples at concentrations of 0.00, 0.25, 0.50, and 1.00 wt.%. Part of the amounts from each obtained type of CaP + Gr admixtures were further blended with *Luffa cylindrica* fibers (Lu), purchased from local suppliers. The fibers’ mass ratio was determined as 14% of the CaP + Gr admixture mass.

Both compact 3D products (CaP + Gr) and porous 3D products (CaP + Gr + Lu) were framed by the isostatic pressing of the respective admixtures (2 sample sets for the compact products and 1 sample set for the porous products—for each Gr concentration) in cylindrical molds (Φ 10 mm) at 2.5 MPa (WK 50 FH PRO, Bernardo, Linz, Austria). Half of the obtained compact samples and all porous samples were further subjected to a thermal treatment (1200 °C/8 h) in air atmosphere (electric furnace, Nabertherm GmbH, Liliethal/Bremen, Germany). Since the Lu fibers’ role is to generate the porous architecture with interconnected channels as result of calcination/combustion (by the sacrificial template method), no relevant results would have been revealed by the investigation of as-admixed and compacted porous products.

All samples were grinded on abrasive SiC paper (P 600–2500) to obtain plane-parallel sample surfaces. The codification of the products was set by the preparation and processing stages, as presented above: the Gr admixing into the ceramic matrix (i.e., CaP + Gr as-admixed), the absence/addition of Lu fibers (i.e., compact/porous products), and the thermal treatment as 1200 °C/8 h indication (i.e., CaP + Gr ± Lu 1200 °C/8 h).

### 2.2. Physico-Chemical Characterization

#### 2.2.1. X-ray Diffraction Analysis

The crystalline quality and phase composition of the specimens was examined by X-ray diffraction (XRD), in Bragg–Brentano geometry. A Bruker D8 Advance diffractometer (Bruker AXS Advanced X-ray Solutions GmbH, Karlsruhe, Germany), with Cu K_α_ (λ = 1.5418 Å) radiation, equipped with a high efficiency LynxEye^™^ linear detector, was used. The XRD diagrams were recorded in the 2θ angular range 10–55°, with a step size of 0.02°, and an acquisition time of 1 s/step. The average over possible compositional in-homogeneities was achieved by the rotation of the samples during analysis with a speed of 30 rpm. The lattice parameters, average crystallite sizes, and phase composition of the samples were extracted by Rietveld whole powder pattern fitting [34], performed with the MAUD v2.31 software.

#### 2.2.2. FTIR-ATR Spectroscopy Measurements

The chemical structure of samples was complementary investigated by Fourier Transform Infra-Red (FTIR) spectroscopy in attenuated total reflectance (ATR) mode. The FTIR-ATR spectra were collected in the fingerprint 1400–550 cm^−1^ wave numbers range using a PerkinElmer BX Spectrum II apparatus (PerkinElmer Corporation, Waltham, MA, USA), equipped with diamond-zinc selenide Pike MIRacle (PIKE Technologies, Madison, WI, USA) ATR crystal attachment (with a diameter of 1.8 mm). The spectra were acquired at a resolution of 4 cm^−1^. A total of 32 scans were recorded for each sample and the resulting interferogram was averaged.

#### 2.2.3. Morpho-Compositional Evaluation

The morpho-compositional evaluation of the Gr reinforced ceramic bodies was performed by scanning electron microscopy (SEM) with a Philips XL 30 ESEM TMP microscope (FEI/Phillips, Hillsboro, OR, USA) coupled with an auxiliary EDAX Sapphire UTW energy dispersive spectrometer (EDAX Inc., Mahwah, NJ, USA). The micrographs were acquired at an acceleration voltage of 25 kV and a working distance of 10 mm [35]. The analyses were performed in five randomly chosen areas.

#### 2.2.4. Mechanical Performance Assessment

The compression strength of both as-admixed and thermally-treated products was tested using a universal test machine Walter + Bai AG, Loehningen (Schaffhausen, Switzerland), type LFV300. The used test speed was 1 mm/min with an acquisition rate of 0.01 s. The measurements were performed in triplicate.

## 3. Results and Discussion

### 3.1. XRD Analysis

**Figure 1:**
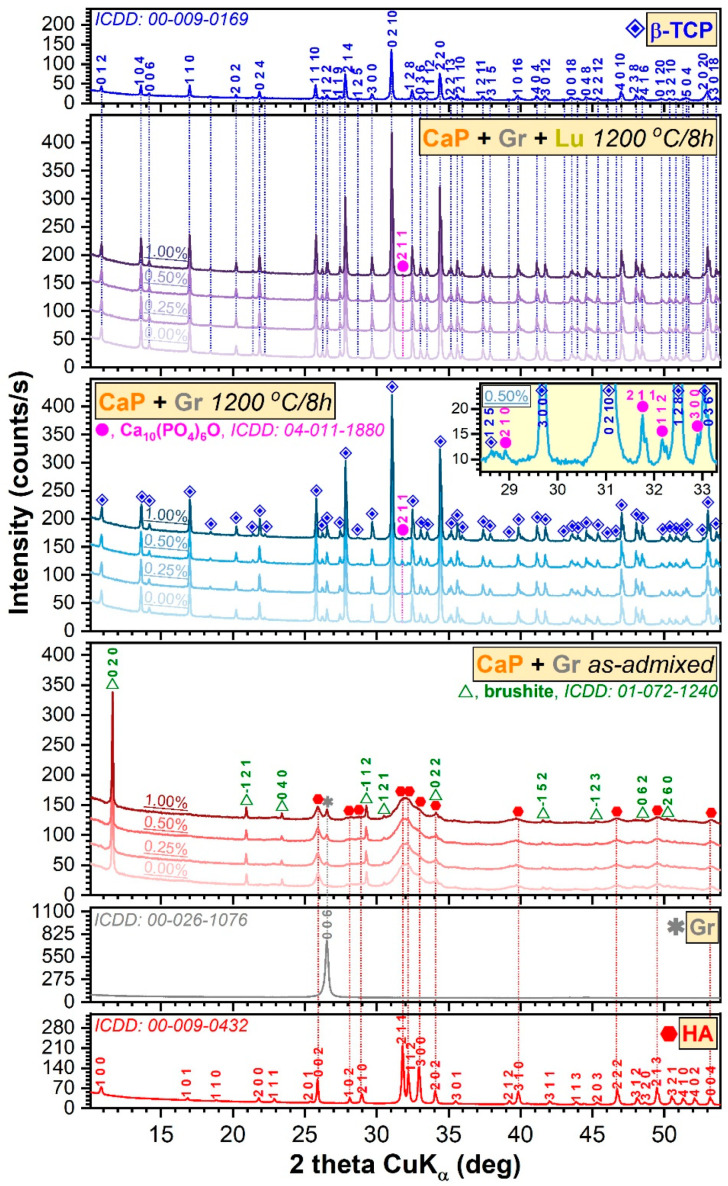
The XRD patterns of the simply ad-mixed and thermally processed samples (with and without *Luffa* fibers) with respect to the reference diffractograms of pure hydroxyapatite, beta-tricalcium phosphate, and graphene grade M powders.

The XRD patterns of the simply ad-mixed and thermally processed samples are presented with respect to the reference diffractograms of pure HA (National Institute of Standards and Technology, Standard Reference Sample (NIST-SRM) 2910b), β-TCP (Sigma-Aldrich, St. Louis, MO, USA)) and Gr, in Figure 1. The source powder consisted of a mix of two phases: a highly crystalline brushite (ICDD: 01-072-1240) and a nanocrystalline HA (ICDD: 00-009-0432). Brushite had a share of ~12 wt.% and an average crystallite size of ~330 nm, whilst nanoHA had a weight of ~88 wt.% and an average crystallite size of ~13 nm. The intentional Gr concentration increase in the samples was supported by the incremental intensification of its corresponding 006 peak (ICDD: 00-026-1076). The thermal processing of the ad-mixed bioceramic samples had led to their structural conversion into a β-TCP phase (ICDD: 00-009-0169). The persistence of the β-TCP at temperatures > 1125 °C [36] suggest a higher stability of this compound due to its doping with (Mg) traces [37], present in the source material (i.e., marble). 

Supplemental, in the case of the thermal treated samples, the presence of low amounts of a secondary phase (see inset of Figure 1) was detected, whose diffraction peaks best matched an oxyapatite-type phase (OA, Ca_10_(PO_4_)_6_O, ICDD: 04-011-1880). The presence of OA is not unprecedented [38], being a result of the HA dehydration (i.e., loss of its structural hydroxyl groups upon heating in air ambient). The concentration of the OA phase never exceeded ~4 wt.%, and its content did not show an obvious dependence on any processing parameter. The β-TCP phase had similar crystalline quality (the full-width at half maximum of the 0 2 10 peak was situated in the narrow range of 0.030–0.33°) irrespective of the initial composition of the samples. The Rietveld analysis unveiled an average crystallite size of ~390 nm in the case of the β-TCP phase. No Gr or graphene oxide peaks were detected at the sensitivity limit of the employed equipment. Similarly, no peaks were signaled in the angular range appertaining to lignocellulosic crystalline phases, which can result by the combustion of Lu fibers [8,39]. Thus, the complete decomposition and elimination of the natural fibers (the porogen agent) and Gr (the reinforcing agent) during sintering can be assumed.

In a previous study, dedicated to the characterization of Gr free sintered marble-derived CaP products blended with Lu fibers, a HA/β-TCP ratio of ~20/80 was obtained [25]. It is thus suggested that in the presence of Gr, a thermal gradient is generated due to its higher thermal conductivity (3000 W/m·K, according to the technical data sheet), with respect to the bioceramic counterpart (1.1–1.25 W/m·K) [40,41,42], leading to an almost complete decomposition into β-TCP, as suggested in Ref. [42].

Therefore, the main influence of the Gr admixing in the ceramic matrix resides in the conversion of the HA/brushite blend into a β-TCP dominant phase (~96 wt.%) with oxyapatite as minuend.

### 3.2. FTIR-ATR Spectroscopy Measurements

**Figure 2:**
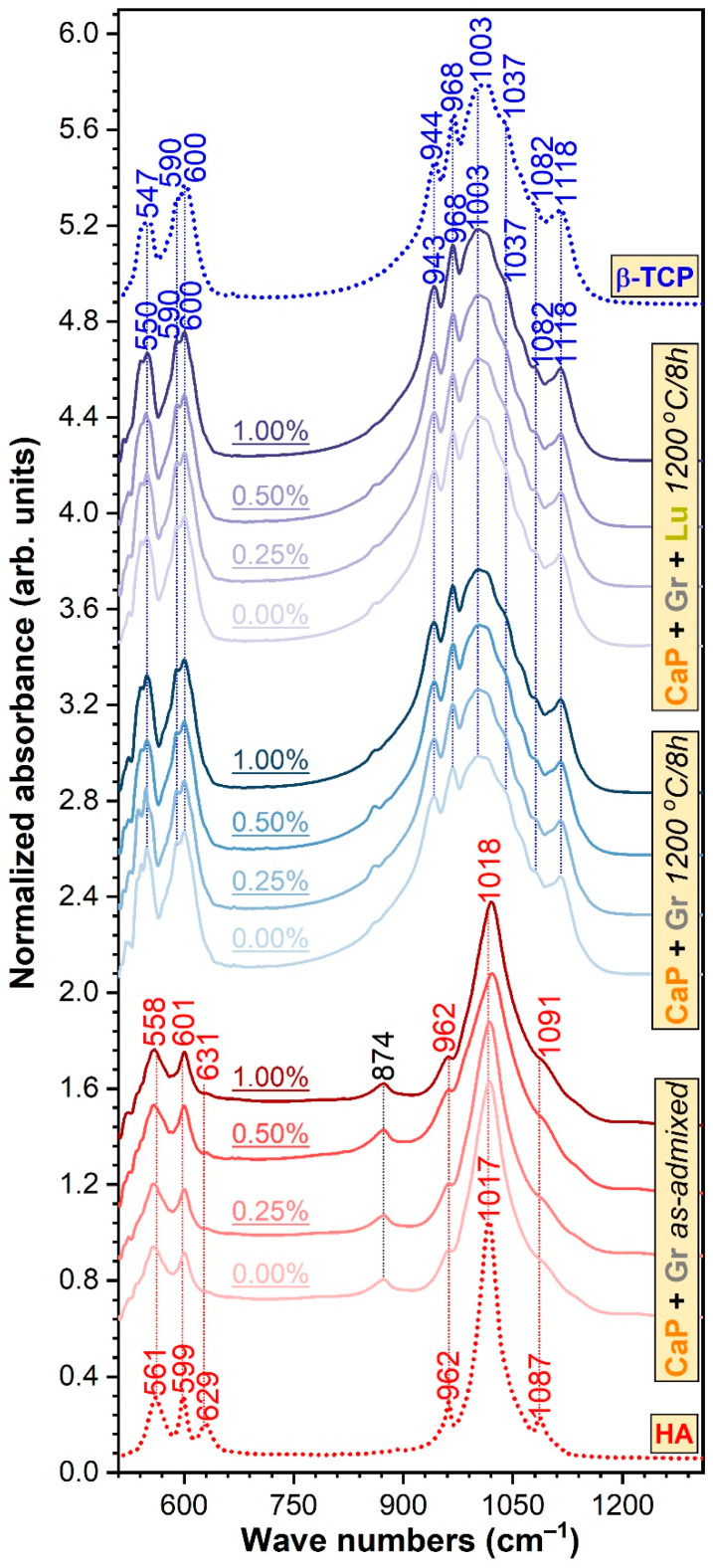
The FTIR-ATR spectra of the simply ad-mixed and thermally processed samples (with and without *Luffa* fibers) with respect to the reference spectra of pure hydroxyapatite and beta-tricalcium phosphate powders.

The FTIR-ATR (Figure 2) bands positioned at the lower wave numbers (~601–600, 590, and 558–547 cm^−1^) are ascribed to the ν_4_ triple degenerated bending vibration modes of the orthophosphate units in CaP phases [43,44,45,46]. In the 1200–900 cm^−1^, The wave numbers region is presented the characteristic ν_1_ non-degenerated symmetric (~962 and 944–943 cm^−1^) and ν_3_ triple degenerated asymmetric (~1118, 1091–1082, 1037, 1018–1003, and 968 cm^−1^) bands of orthophosphate groups in CaPs [43,44,45,46]. The presence of the brushite phase, in the case of the simply ad-mixed samples, was suggested by the maximum at ~874 cm^−1^, which can be linked to the P–O(H) stretching vibrations in the (HPO_4_)^2−^ groups [47]. The partial juxtaposition in this region of the ν_2_ symmetric stretching of carbonate groups cannot be excluded [43]. The feeble shoulder peaking at ~631 cm^−1^, present in the case of the ad-mixed samples only, is associated to the libration vibrations of structural hydroxyl groups in HA [43]. Its low intensity is descriptive of nano-crystalline HAs, and it is determined by the reduced crystallite size and the consequent distortions and atomic disorder in the HA lattice, which hinders the incorporation of OH^−^ groups [48,49,50]. The absence of the OH^−^ libration band in the case thermally processed samples denoted their de-hydroxylation, in good agreement with the XRD data (which unveiled the presence of the β-TCP and OA phases only). The formation of β-TCP was supported by the characteristic intricate allure of the IR spectral envelopes of the thermally processed specimens, and high similarity to the spectrum of the pure commercial β-TCP compound, used as reference sample. The HA–β-TCP discrimination can also be made by the incidence of the three trademark IR vibration band of β-TCP: ν_1_ symmetric stretching band centered at ~944–943 cm^−1^ and the ν_3_ asymmetric stretching bands peaking at ~1118 and 968 cm^−1^ [44].

### 3.3. SEM/EDS Evaluation

**Figure 3:**
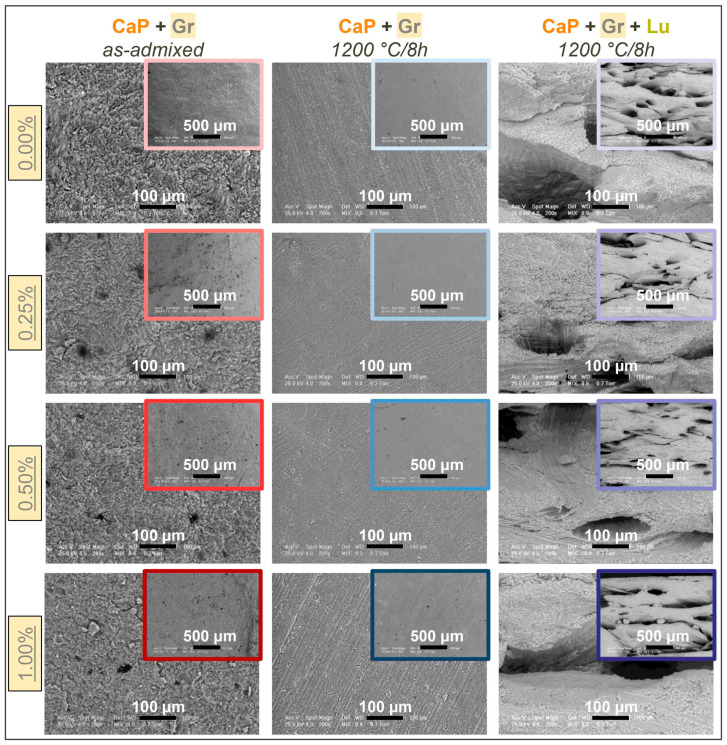
Representative SEM images of CaP + Gr as-admixed and thermally-treated CaP + Gr ± Lu 3D products acquired at two magnifications for each Gr amount.

**Figure 4:**
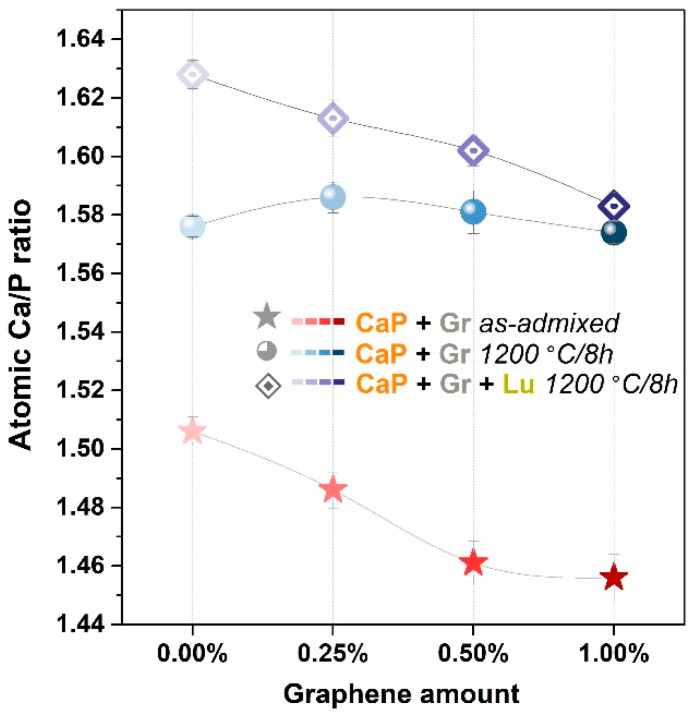
Evolution of the Ca/P atomic ratio for the as-admixed CaP + Gr and the thermally-treated CaP + Gr ± Lu products, at each Gr amount.

The morphological and compositional evolution of as-admixed and thermally-treated compact (CaP + Gr) and porous (CaP + Gr + Lu) products is displayed in Figure 3 and Figure 4, respectively. The surface morphology of the compact products Figure 3, prior to sintering, depicts a compact microstructure, with fine-edged and nano-sized polyhedral grains of the CaPs matrix and few residual micropores. The dark-rounded-spots appearance corresponds to the Gr nanoplatelets distribution in the ceramic powder, which exhibits a relatively increased tendency to agglomerate only at lower Gr concentration. This tendency is generally a direct effect of the graphene’s 2D geometry and layer interaction with other particles [51]. Further, this is an important aspect since the dispersion level of the reinforcement phase in the ceramic matrix is a key-factor for defining and attaining the required mechanical features [52].

After sintering, a more pronounced compacted aspect and densification with homogenously interlinked grains and indistinguishable grain-shapes was induced by the coalescence of the CaP particles. Moreover, the Gr incorporation into the ceramic matrix led to the formation of a nano- and micro-scaled porosity at high temperature, while its presence got extinct, in accordance with the XRD and FTIR-ATR spectroscopy findings. The beneficial effect of Gr as reinforcement agent on the bioceramic ultrafine microstructure is primarily based on the compatible small sized nanoplatelets which continuously collide with the CaP particles during mixing stages [28,42,53]. Secondly, the presence of Gr was reported also as strengthening factor through grain size refinement during ceramic materials sintering [30]. This comes in agreement with the micrographs presented in Figure 3, regardless of the Gr elimination in Figure 1 and Figure 2.

Further, a gradually increased porous character in accordance with the admixed Gr amount, in the case of compact products, was depicted by the SEM inspections. This is both desired and associated with an improved osteoblast cells adherence and proliferation [3,42], especially when compared to similar samples, with no matrix reinforcement [20].

In regard to thermally-treated porous products Figure 3, the micrographs confirmed the complete removal of the Lu fibers and their ability to form bone-like fibro-vascular arrays with micro-sized internal channels and secondary micro-porous surfaces induced/impregnated inside the channels walls, as result of intensive decomposition processes of the organic matter and volatiles expulsion during the high temperature treatments [54].

Hence, these results validate the reproducibility of the fibers incorporation method and sintering behavior, as previously investigated in Refs. [8,25]. Furthermore, the influence of Gr addition reveals an evolution from mostly round micro-pores/channels (0.25–0.50 wt.% concentrations) to spherical and flattened pores/channels at maximum Gr amount (1.00 wt.%). Moreover, incipient cracking and large ceramic particles disruptions along the formed channels were also observed at 1.00 wt.% Gr, which are prone to destabilize the 3D structure when implanted. Therefore, the reinforcement of the ceramic matrix for porous products can be performed with specific limitations (i.e., maximum 0.50 wt.% Gr) in view of bone-mimicking templates with mechanical resistance. The presence of round micropores with compatible sizes (i.e., in the range of Haversian canals: 50–90 μm in diameter, and higher) and associated secondary micro-porosity expand the surface area favorable for cells adhesion and proliferation, nutrients transport, and blood vessels and nerves hosting [3,55]. However, the optimum pores shape and geometry are two aspects still under debate in regard to the development of vascular networks. It was revealed that lamellar or flat pores/channels structures are still fit as long as they fulfill the size requirements for cells migration and proliferation (in Figure 3 all pores diameters exceed 100 μm up to 0.50 wt.% Gr). In some cases, they were found to induce better angiogenesis and faster bone formation and mineralization when implanted, as compared to spherical shaped pores [56].

The EDS evaluation outlined similar trends for both as-admixed and thermally-treated products. The resulting Ca/P atomic ratios in Figure 4 were in agreement with the drastic decomposition of HA after sintering, evidenced by XRD and FTIR-ATR spectroscopy in Figure 1 and Figure 2, respectively.

The Ca/P ratios for as-admixed and thermally-treated compact products pinpointed a transition from lower values range induced by the presence of brushite phase (Ca/P = 1) [57] to the β-TCP dominant phase ones (1.57–1.59) [25,26]. However, the calculated values for the porous products indicated a slightly higher variation (situated in the 1.59–1.63 range), inversely proportional with the Gr amount, suggesting that the Lu fibers could also act as a compositional adjuvant and mediator. The high temperature decomposition of HA/brushite into β-TCP, directly correlated to lower Ca/P ratios (<1.67) and structural strength, is often reported in the literature [26,42,58].

### 3.4. Mechanical Performance Assessment

**Figure 5:**
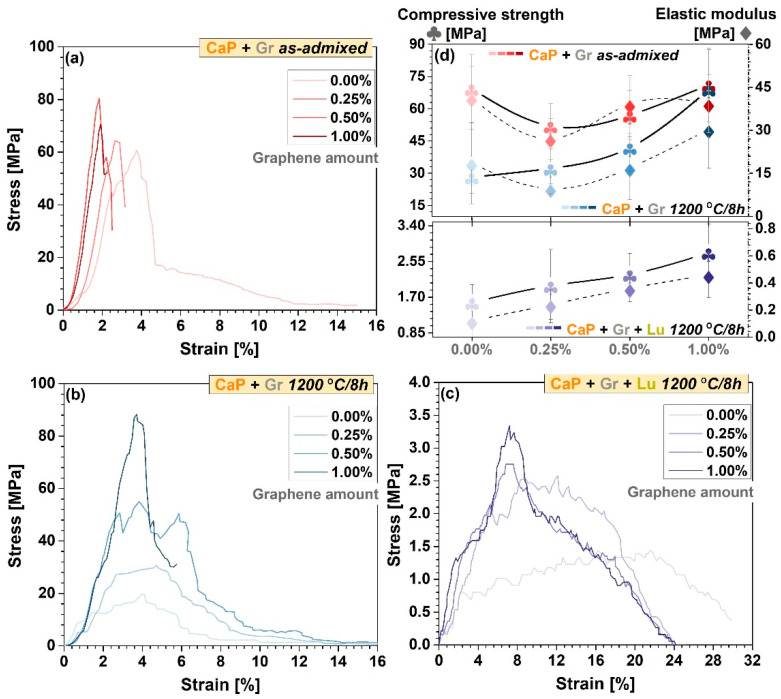
(**a**–**c**) Characteristic stress–strain curves and (**d**) comparative compressive strength and elastic modulus for CaP + Gr as-admixed and CaP + Gr ± Lu thermally-treated products, at each Gr amount.

The stress–strain curves acquired during uniaxial compression testing of as-admixed and thermally-treated CaP + Gr samples (compact products) and thermally-treated CaP + Gr + Lu (porous products) are comparatively presented in Figure 5a–c. The subtracted mean values of the compressive strength and elastic modulus and corresponding standard deviation results are graphically represented in Figure 5d.

Prior to sintering, the aspect of the stress–strain curves indicates overall fragility (accentuated steep slopes) with the gradual increment of the Gr amount incorporated into the ceramic matrix (Figure 5a), which led also to an irregular alternation of high and low values of the compressive strength with an incrementing factor of ~0.75–1.03, as reported to Gr-free samples Figure 5d.

Of greater interest for biomedical applications, this mechanical performance pattern changed completely and favorably after sintering. The thermally-treated samples presented wider or narrower plateau-areas as a result of improved elastic modulus or possible internal cracks propagation, which tend to confine and even become extinct, with the increase of Gr concentration. Although the thermally-treated samples seem to achieve lower compressive strength values as compared to the as-admixed ones, they outlined a constant, linear ascending trend line by ~1.14–2.45 according to the Gr reinforcing amount, in contrast to the Gr-free ones. Moreover, at 1.00 wt.% Gr the steep slope aspect of the stress–strain curve in Figure 5b indicates improved mechanical features in agreement with the highest obtained compressive strength values (Figure 5d). The attained values range is compatible with the mechanical requirements for proper bone reconstruction [20,59]. Therefore, the Gr phase acts a crack-barrier improving the fracture resistance and augmenting the compressive strength of both type of products. This comes in close relation to Gr ability to absorb more stress at surface level, through the electrostatic positive-charged Gr-CaP interactions and favorable interfacial bonding with the ceramic particles [26,53]. The crack-barrier positive effect of Gr has been previously identified also by other authors [28,51].

For compact products, the stress–strain curves highlighted the overall reinforcing effect of the Gr particles, proportional with used concentrations. Gr presence underlined the reduction of strain at the maximal load, which was found in close correlation with certain improvements in modulus absolute value. In this context the improved rigidity of the samples can be explained by the favorable interaction between Gr and CaP particles. The sintering process without doubt can bring improvements in this respect. However, the high temperature of the sintering process, which improves the particles interaction, can affect the Gr stability. Cleavage effects can appear between graphenic layers, and therefore a small decrease in the mechanical properties was registered also for the sintered (versus as-admixed) samples, as presented in Figure 5d.

The further incorporation of Lu fibers and high-temperature sintering led to a more ductile behavior of the 3D products, with a similar trend for enhanced compressive strength values by ~1.25–1.76 and decreased deformation tendency, in accordance with the increased Gr amount. However, compared to the thermally-treated compact products, the compressive strength values registered a sharp decline by approximately 96%. This contrasting behavior is determined by the elevated level of pores/channels sizes and overall porosity (Figure 3), directly responsible for a reduced mechanical strength as compared to pure HA [60] or compact structures [3,53]. In the case of porous samples, despite a certain decrease of the overall mechanical strength of the analyzed samples, Lu fibers can change more the stress-strain profiles regardless of the Gr amount. In good agreement with the previously presented SEM data (Figure 3), the pores presence can play a particular role. In the sintering process, the expansion during gas phase elimination, assures the supercritical conditions (locally) to improve the material local plasticity determining a certain orientation on the pore walls. This state allowed for a different takeover of the load and stress during crack propagation. In this context, when comparing Figure 5b with Figure 5c, the plateau region from the stress–strain curves highlights a less rigid behavior when Lu fibers were used.

It is well-known that the overall strength of the natural bones is governed by bone dimensions (i.e., short/long), structure, shape, internal architecture (i.e., cortical/trabecular), and decreases in the area where an implant was chosen for reconstruction [61]. Moreover, the requirements for an implant diverge depending on the intended role (e.g., replacement, reinforcing, treatment) [26], yet for short- and also long-term support, they should fulfill all criteria regarding the mechanical performance and the mimicking architecture of the host tissue, and the compositional stability in the physiological environment [26,62].

In this regard, another important parameter for the products’ prospective behavior is the elastic modulus. Any mismatch at the bone-implant interface, especially increased values with respect to those of the natural bone, could induce stress shielding or could implicate a risk of implant failure [26]. In this view, the incorporated Gr acted as a mechanical adjuvant, with incrementing factors of ~1.6–3 and ~1.3–3.8 for the compact and porous products, as compared to the Gr-free samples, yet in the customary reported limits of cortical and trabecular bone [26,63]. For the porous products, the distinctive lower values followed the same trend line as in the case of the corresponding compressive strength.

To sum up, the designed 3D reinforced and sintered products comply with the mechanical requirements of the bone reconstructive field, here including both cortical and trabecular sites [64]. Even though the strength of thermally-treated porous and compact products are comparable, they are still compatible for non- and load-bearing applications—for compact products that exceed the lower limit of the cortical bone resistance (20 MPa) [20,59]. Moreover, when needed, the mechanical and architectural features can be adapted by tuning the reinforcement (Gr) and porogen (Lu fibres) agents’ ratio.

## 4. Conclusions

Compact and porous products were successfully fabricated from naturally derived calcium phosphates, using *Luffa* fibers as porous template and graphene as mechanical reinforcement agent, by a one-stage binder/additive-free preparation method.

The adequate dispersion and incorporation of the two non-ceramic materials by mechanical processing into the ceramic matrix preserved its initial structural composition, but induced a more pronounced decomposition of the HA/brushite blend into β-TCP dominated compound upon high temperature sintering (1200 °C/8 h). Even so, the incorporation of Gr did not influence the stability of the β-TCP dominated biphasic product. 

The morphological and mechanical evaluation of the 3D products confirmed: (i) the benefits of adding Gr, with favorable consequences upon the micro-porosity of the compact products, and the global improvement of compressive strength and elastic modulus for both type of sintered products (compact and porous), and (ii) the *Luffa* fibers preserved ability, also in the current context, to generate vascular-like-arrays with interconnected pores/channels, while acting as both compositional adjuvant and mediator.

Based on the toughening mechanism trend induced by Gr, it is suggested that the Gr amount should be restricted to a maximum 0.50 wt.% in the case of sintered porous structures, in order to reduce the risk of internal cracking along the channel walls and distinct pores deformation. On long-term premises, both products can be considered for the production of *bone regenerator sites* with tunable compositional, architectural, and mechanical features.

## Data Availability

All data generated or analyzed during this study are included in this published article. The raw data can be made available from the authors upon reasonable request.
